# Establishment and characterization of a novel lymphangiosarcoma cell line (MO-LAS) compared with the hemangiosarcoma cell line (ISO-HAS)

**DOI:** 10.1002/cam4.12

**Published:** 2012-07-13

**Authors:** Mikio Masuzawa, Mamiko Masuzawa, Yuhko Hamada, Nobuko Arakawa, Mari Mori, Masako Ishii, Shigeo Nishiyama

**Affiliations:** 1 Department of Regulation Biochemistry, Kitasato University School of Allied SciencesSagamihara, Japan; 2 Department of Dermatology, Kitasato University School of MedicineSagamihara, Japan

**Keywords:** Cell line, hemangiosarcoma, lymphangiosarcoma, *PROX1*

## Abstract

The concept of “lymphangiosarcoma” remains obscure. Therefore, we reported a patient with lymphangiosarcoma, resistant to immunotherapy. The patient presented with impressive and discriminative features: clinically an ill-defined edematous lesion with lymphorrhea and pathologically atypical vascular channel formation without extravasation of blood, clearly distinguished from common angiosarcoma with hemorrhage. From this case, a lymphangiosarcoma cell line, MO-LAS, was established and its characteristics were compared with the hemangiosarcoma cell line, ISO-HAS. Flow cytometric analysis revealed that MO-LAS was negative for factor VIII-related antigen, but positive for CD31, D2-40, NZ-1, and vascular endothelial growth factor receptor-3 (VEGFR-3), similar to ISO-HAS. However, MO-LAS expressed a much higher level of homeobox gene *PROX1*, indicating a lymphatic phenotype, compared with ISO-HAS. Furthermore, MO-LAS showed a much lesser expression of oncogenes and much lower sensitivity against lymphokine-activated killer (LAK) cells. Lymphangiosarcoma may be difficult to recognize by the immune system. Conclusively, the establishment of MO-LAS, a novel angiosarcoma cell line bearing lymphatic characters, strongly suggests the entity of lymphangiosarcoma.

## Introduction

The concept of “lymphangiosarcoma” remains obscure. Therefore, we reported a patient with lymphangiosarcoma, resistant to immunotherapy. The patient presented with impressive and discriminative features: clinically an ill-defined edematous lesion with lymphorrhea and pathologically atypical vascular channel formation without extravasation of blood, clearly distinguished from common angiosarcoma with hemorrhage. From this case, a lymphangiosarcoma cell line, MO-LAS, was established and its characteristics were compared with the hemangiosarcoma cell line, ISO-HAS. Flow cytometric analysis revealed that MO-LAS was negative for factor VIII-related antigen, but positive for CD31, D2-40, NZ-1, and vascular endothelial growth factor receptor-3 (VEGFR-3), similar to ISO-HAS. However, MO-LAS expressed a much higher level of homeobox gene *PROX1*, indicating a lymphatic phenotype, compared with ISO-HAS. Furthermore, MO-LAS showed a much lesser expression of oncogenes and much lower sensitivity against lymphokine-activated killer (LAK) cells. Lymphangiosarcoma may be difficult to be recognized by the immune system. Conclusively, the establishment of MO-LAS, a novel angiosarcoma cell line bearing lymphatic characters, strongly suggests the entity of lymphangiosarcoma.

The concept of “lymphangiosarcoma” was first developed in 1948 by Stewart and Treves who reported six cases of a malignant vascular tumor occurring in chronic lymphedema of the upper extremity after mastectomy [1]. It is based on the presumption of lymphatic differentiation in postoperative lymphedema. After this report, a number of reports used the appellation of lymphangiosarcoma without clear definition [2–4]. The concept of lymphangiosarcoma has given rise to the confusion. As a consequence, malignant vascular tumors have been categorized as a single entity, angiosarcoma [5], because it was difficult to distinguish whether each angiosarcoma was derived from a blood vessel or a lymph vessel. Nowadays, the presence of lymphangiosarcoma has been reviewed mainly from a pathological aspect by the appearance of numerous lymphatic markers [6–14]. However, the results of these studies have been unable to provide definitive conclusions.

In this study, we advance the clinical and histopathological characters of lymphangiosarcoma based on our experience of 71 patients with angiosarcoma at the Kitasato University Hospital [15]. Furthermore, a novel angiosarcoma cell line, derived from a typical case of lymphangiosarcoma, was established; and its characteristics are compared with the hemangiosarcoma cell line, ISO-HAS [16], already established in our laboratory. The final aim of this study was to confirm an entity of lymphangiosarcoma.

## Materials and Methods

Informed consent was obtained from the patient and family prior to conducting this study, and the Ethics Committee of the Kitasato University Hospital approved the study.

### Case history

A 77-year-old man was admitted to the Kitasato University Hospital on 16 June 1993, because of an ill-defined edematous lesion covering the scalp and face and spreading from the last 10 months. Histological examination confirmed angiosarcoma. He was treated with intralesional and intra-arterial administration of recombinant interleukin-2 (rIL-2) (Shionogi, Osaka, Japan) and LAK cells. Total 108 million units of rIL-2 with 2.3 × 10^10^ LAK cells were administered, but the disease was progressive and caused pleural, peritoneal, and cardiac effusions. The patient died on 6 March 1994. The autopsy revealed whole body metastasis including lung, liver, spleen, adrenal gland, vertebrae, rib, pleura, diaphragm, and lymph node without any hemorrhage.

### Cell cultivation

The pleural fluid harvested from the case was centrifuged at 1500 rpm for 10 min, then cells in the pellet were cultured with angiosarcoma cell-culture medium [16] composed with high-glucose Dulbecco's modified Eagle's medium (Gibco BRL, Grand Island, NY), supplemented with 10% (v/v) heat-inactivated fetal calf serum (FCS) (JRH Biosciences, Lenexa, KS) and 50% (v/v) conditioned cultured medium of a murine angiosarcoma cell line (ISOS-1) [17] at 37°C in a 5% CO_2_ incubator. Cell morphology was monitored under a phase-contrast microscopy (Olympus, Tokyo, Japan). The doubling time was calculated from the exponential-growth phase. As a control, a human hemangiosarcoma cell line (ISO-HAS) [16] was cultured with angiosarcoma cell-culture medium, and normal human umbilical vein endothelial cells (HUVEC) (Kurabo, Tokyo, Japan) and neonatal normal human dermal lymphatic microvascular endothelial cells (HMVEC-dLy Neo) (Takara-Bio, Shiga, Japan) were also cultured with the medium provided by the manufacturer.

### Immunohistochemistry

The 4-μm-thin sections were prepared from tissue fixed with 10% formalin. The first antibody was anti-*PROX1* (1:100 dilution; Abcam, MA) and then EnVision™ (Dako, Tokyo, Japan) was used for the second antibody, visualized by 3-amino-9-ethylcarbazol.

### Flow cytometric analysis

Single-cell suspensions (1 × 10^6^ cells/mL) were ready for flow cytometric analysis on a FACScan (Becton Dickinson, Mountain View, CA). Antibodies were prepared as follows. The first monoclonal antibodies: anti-factor VIII-related antigen (1:50 dilution; Dako), anti-CD31 (1:40 dilution; Dako); anti-D2-40 (1:100 dilution; Nichirei, Japan) and the second antibody: AlexaFluor 488 labeled Goat anti-Mouse IgG (1:100 dilution; Molecular Probes, OR); anti-podoplanin (NZ-1) (1:500 dilution; Sigma Aldrich, St. Louis, MO), the second antibody: biotin-conjugated anti-Rat IgG (1:1000 dilution; Cappel, Aurora, OH), and the third antibody: fluorescein isothiocyanate-conjugated streptavidin (1:100 dilution; Cappel); anti-VEGFR-3 (1:100 dilution; Abcam) and the second antibody: AlexaFluor 488 labeled Goat anti-Rabbit IgG (1:100 dilution; Molecular Probes). The usage of each antibody was according to the instruction manuals.

### LAK cell preparation

Preparation of LAK cells followed the techniques described previously [18]. Briefly, peripheral blood lymphocytes (PBL) were obtained using Ficoll-Hypaque (Pharmacia Fine Chemicals, Uppsala, Sweden) density centrifugation of whole heparinized blood from healthy volunteers in our laboratory. Culture flasks (Falcon; Becton Dickinson, Franklin Lakes, NJ) were immobilized with 5 μg/mL of OKT-3 (Kyowa Hakko Kogyo, Tokyo, Japan) at 37°C for 1 h and washed with phosphate-buffered saline (PBS) twice before the addition of cells. PBL were cultured with RPMI 1640 medium (Gibco) containing 10% FCS, 50 mg/mL of Gentamycin (Gibco), and 200 IU/mL of rIL-2 on the immobilized culture flasks for 1 week. Then the cultured cells were transferred to nonimmobilized culture flasks and culturing was continued. Medium was replaced every 3–4 days with fresh medium containing rIL-2. After 2 weeks, the cells were washed twice with PBS and used for in vitro cytotoxicity assay.

### Cytotoxicity assay

Cytotoxicity assay followed the techniques described previously [18]. Briefly, 1 × 10^6^ target cells were labeled with 100 mCi of Na_2_CrO_4_^51^Cr (Japan Isotope Association, Tokyo, Japan) for 60 min at 37°C. The cells were washed and adjusted to 5 × 10^4^ cells/mL and distributed in 100-μL aliquots to 96v-microwell plates containing 50 μL of dilutions of effector LAK cells that lead to appropriate effector-to-target (E:T) ratios. The plates were centrifuged at 500 rpm for 5 min, and then incubated for 4 h in a CO_2_ incubator. Aliquots of the supernates were collected, and the amount of ^51^Cr released was counted in a gamma counter. The percentage of spontaneous release (spontaneous cpm/total cpm) was <15% in all instances. The percentage cytotoxicity was calculated by the following formula: % cytotoxicity = (*E* – *S/T* – *S*) × 100, where *E* = experimental release (cpm); *S* = spontaneous release (cpm); and *T* = total incorporated release (cpm).

### Reverse transcription-polymerase chain reaction (RT-PCR) analysis

The mRNA expression of *p53*, *c-jun*, *c-fos,* and *c-myc* oncogenes was analyzed using RT-PCR. Total cellular RNA was extracted from each cell line using ISOGEN (Nippon Gene, Tokyo, Japan) following the manufacturer's instruction, complementary DNA (cDNA) was synthesized from 1 μg of total RNA using High-Capacity cDNA Reverse Transcription Kit (Applied Biosystems, Foster City, CA). cDNA was used for PCR with primers. The primers used were: *p53*; forward, 5′-CTGAGGTTGGCTCTGACTGTACCACCATCC-3′ and reverse, 5′-CTCATTCAGCTCTCGGAACATCTCGAAGCG-3′, *c-jun*; forward, 5′-GCATGAGGAACCGCATCGCTGCCTCCAAGT-3′ and reverse, 5′-GCGACCAAGTCCTTCCCACTCGTGCACACT-3′, *c-fos*; forward, 5′-CCCGCAGACTCCTTCTCCAGCATGGGC-3′ and, reverse, 5′-GGCTGCAGCCATCTTATTCCTTTCCCT-3′, *c-myc*; forward, 5′-TACCCTCTCAACGACAGCAGCTCGCCCAACTCCT-3′ and reverse, 5′-TCTTGACATTCTCCTCGGTGTCCGAGGACCT-3′ or β-actin forward, 5′-GTGGGGCGCCCCAGGCACCA-3′, reverse, 5′-CTCCTTAATGTCACGCACGATTTC-3′. The reactions were performed for 30 cycles to obtain linearity in the PCR product. After the PCR, each 10-μL sample was analyzed on a 1.5% agarose gel in Tris-borate-EDTA buffer and stained with ethidium bromide.

### Real-time PCR

Total RNA isolated from ISOGEN was treated with DNase I (Ambion Diagnostics, Austin, TX). One microgram of total RNA was then reverse transcribed into cDNA. Real-time PCR was carried out using iQ SYBER Green Supermix (Bio-Rad, CA), according to the manufacturer's instructions. β-actin was used as a housekeeping gene. The primers used were: β-actin forward, 5′-GAGCGCGGCTACAGCTT-3′, reverse, 5′-TCCTTAATGTCACGCACGATTT-3′, *PROX1* forward, 5′-CCAGCTCCAATATGCTGAAGACCTA-3′, reverse, 5′-CATCGTTGATGGCTTGACGTG-3′ [19]. PCR was performed using CFX96 (Bio-Rad). The quantitative target gene expressions were determined in comparison with the gene expression of ISO-HAS. The relative gene expressions were determined using the comparative critical threshold (DDCT) method.

## Results

### Patient findings

**Figure 1:**
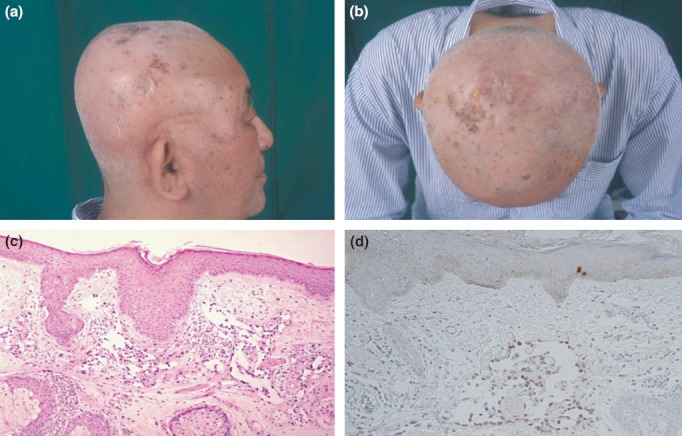
The clinicohistopathological features of a 77-year-old male. (a and b) The whole scalp showed diffuse, ill-defined edematous change with oozing fluid from various locations. (c) Examination of a scalp skin biopsy specimen revealed numerous irregular vascular channels lined with atypical endothelial cells without hemorrhage. (d) Immunohistochemically, the atypical cells were positive for *PROX1*.

**Figure 2:**
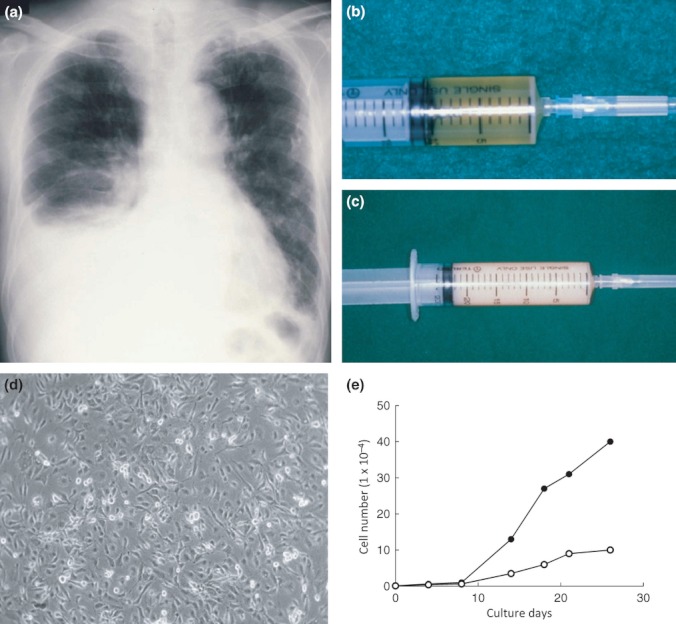
The establishment of the novel cell line MO-LAS. (a) Chest x-ray of hydrothorax, (b) clear yellowish pleural effusion, and (c) milky ascites were harvested. (d) The cell line (MO-LAS) cultured from pleural effusion grew in a monolayer. (e) The cell growth curve in vitro. The conditioned medium of the murine-phenotypic angiosarcoma cell line (ISOS-1) was supplemented in 50% of culture medium (—•—) or not (—○—). The cells required the conditioned medium of ISOS-1 to grow.

The patient had an ill-defined edematous lesion on the whole scalp and face with oozing lymphatic fluid (Fig. 1a and b). Examination of a scalp skin biopsy specimen revealed numerous irregular vascular channels lined with atypical small cells without extravasation of blood (Fig. 1c). Immunohistochemically, the atypical small cells were positive for CD31, D2-40, and *PROX1* (Fig. 1d) but weakly positive for VEGFR-3 and negative for factor VIII-related antigen. Hydrothorax and ascites were detected using x-ray examination (Fig. 2a). The suctioned pleural fluid was yellowish and ascites fluid was milky (Fig. 2b and c). These clinicohistopathological findings without hemorrhage anywhere indicated a diagnosis of lymphangiosarcoma.

### Establishment of cell line and flow cytometric analysis

The harvested cells from the pleural effusion began proliferation 3 weeks later with the angiosarcoma cell medium. After subcloning by the limiting dilution technique, the cells grew well in a Petri dish, and a new cell line was subsequently established and named MO-LAS. The cells grown in a Petri dish were polygonal in shape and grew in a monolayer (Fig. 2d). The growth activity was dependent on the supplement of the murine-phenotypic angiosarcoma cell line (ISOS-1) conditioned medium (Fig. 2e). The doubling time was 91-h using the angiosarcoma cell medium including 50% of ISOS-1 cultured conditioned medium. The cultivation of MO-LAS has been maintained since 1993. To confirm the autologous origin, a Short Tandem Report study was performed between MO-LAS and frozen normal skin tissue from the patient. Results from 10 DNA loci indicated that MO-LAS was genetically identical to the patient.

**Figure 3:**
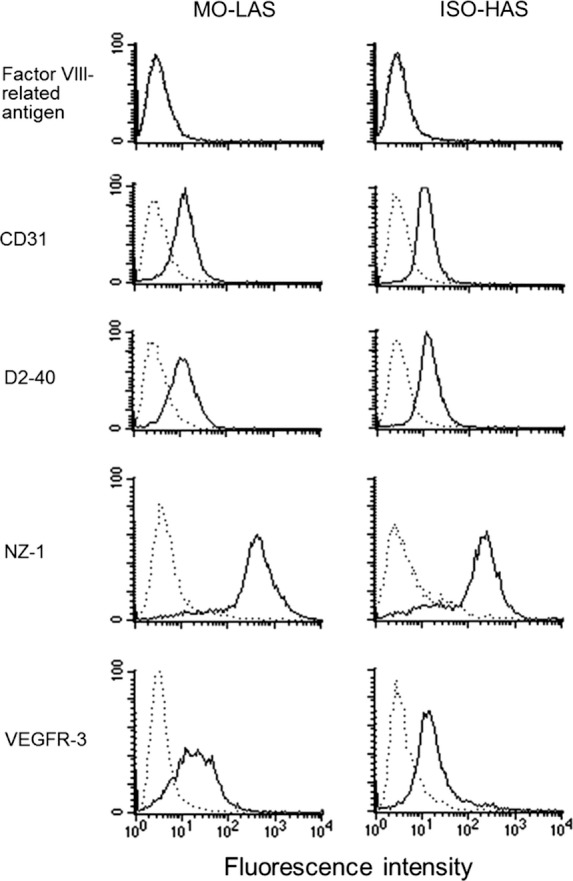
MO-LAS and ISO-HAS were analyzed by fluorescence activated cell sorter (FACS) using vascular markers, including factor VIII-related antigen, CD31, D2-40, NZ-1, and VEGFR-3. Both cell lines showed similar results, which were negative for factor VIII-related antigen, but positive for other markers. MO-LAS has characteristics of angiosarcoma as does ISO-HAS.

To clarify cellular phenotypes, the cells were subjected to flow cytometric analysis using blood vascular and lymphatic makers. The cells were negative for factor VIII-related antigen, but positive for CD31, D2-40, NZ-1, and VEGFR-3, similar to ISO-HAS (Fig. 3).

### Expression of *PROX1* gene in MO-LAS

**Figure 4:**
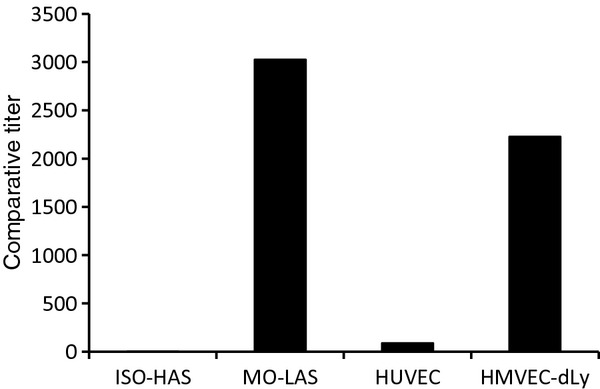
Correlation of the gene expression levels of *PROX1* among MO-LAS, ISO-HAS, HUVEC, and HMVEC-dLy on the base of ISO-HAS level using real-time PCR. MO-LAS expressed gene over 3000-fold of ISO-HAS. As control, the expression of *PROX1* was very low in HUVEC, but was a moderate level in HMVEC-dLy.

To assess the lymphatic-origin of MO-LAS, the expression level of a lymphatic-specific marker, *PROX1*, was measured using quantitative RT-PCR (Fig. 4). *PROX1* in MO-LAS expressed over 3000-fold compared with ISO-HAS. As a positive control, HMVEC-dLy expressed high-level *PROX1* gene, much more than did HUVEC.

### Analysis of oncogenes expression

**Figure 5:**
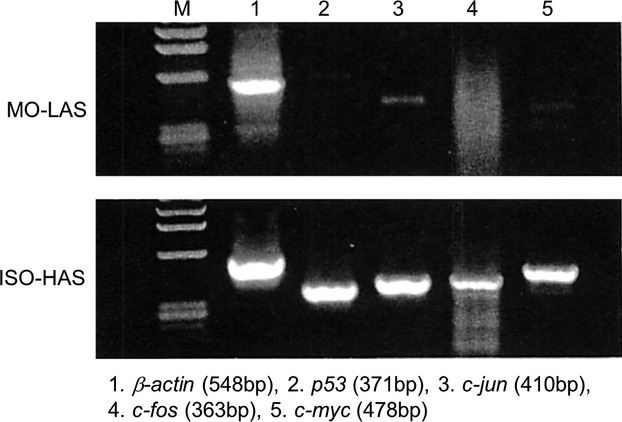
Detection of oncogene mRNAs, such as *p53*, *c-jun*, *c-fos,* and *c-myc*, in MO-LAS and ISO-HAS using RT-PCR. The upper panel shows the RT-PCR product of each oncogene-mRNA in the MO-LAS, whereas the lower panel shows that in the ISO-HAS. The names of the oncogenes are given in the figure. MO-LAS slightly expressed only *c-jun* mRNA, whereas ISO-HAS intensively expressed *p53*, *c-jun*, *c-fos*, and *c-myc* mRNAs.

As Figure 5 shows, MO-LAS slightly expressed only *c-jun* mRNA, whereas ISO-HAS expressed *p53*, *c-jun*, *c-fos*, and *c-myc* mRNAs intensively. The expression level of oncogene mRNAs in MO-LAS confirmed relatively very low compared with these in ISO-HAS.

### Cytotoxicity of cell lines by LAK cells

**Figure 6:**
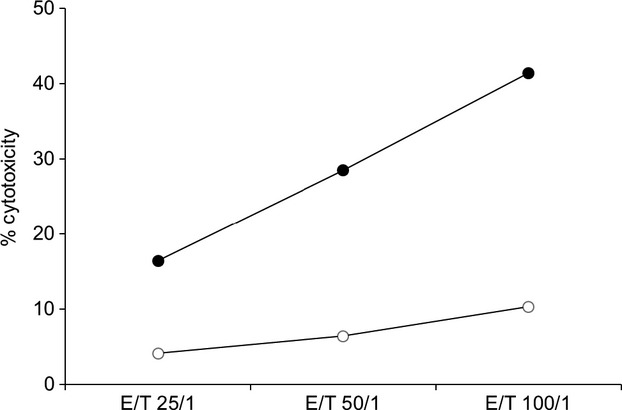
LAK cytotoxicity assay for MO-LAS and ISO-HAS. LAK cells were prepared by cultivation of peripheral blood mononuclear cells of volunteers using rIL-2 and CD3-immobilized flasks. ISO-HAS was moderately sensitive (—•—) to LAK activity according to the E/T ratio, whereas MO-LAS was resistant (—○—).

Immune sensitivity of MO-LAS was studied by reactivity for LAK cells. The LAK cells showed cytotoxicity against ISO-HAS moderately, whereas much less against MO-LAS (Fig. 6). Triple examinations induced the same results.

## Discussion

In this study, a cell line, designated as MO-LAS, was established from the pleural effusion of a patient diagnosed as having angiosarcoma, which we prefer to call lymphangiosarcoma, according to our diagnostic precepts.

The appellation of lymphangiosarcoma has been described without an adequate definition in previous reports [1–4]. However, lymphangiosarcoma was recently suggested as a subset of angiosarcomas by a number of researchers [20–24]. Clinicopathological characteristics of lymphangiosarcoma were reported as occurring on the scalp and face, with prominent hobnailing of tumor cells and lymphoid aggregates [23, 25]. Meanwhile, our clinicopathological concept of lymphangiosarcoma is based on our 12 cases of 71 angiosarcomas [15]. The prominent features are clinically lymphorrhea from ill-defined edematous lesions without bleeding, and pathologically numerous irregular vascular channels lined with atypical hobnailed endothelial cells without extravasation of blood in either the original or metastatic lesions, and, in some cases, lymphoid aggregates. The edematous lesions in our 12 cases, caused by tumorigenic transformation of lymphatics were definitely different from the premonitory lymphedema reported by Stewart and Treves ([1] and others [2–4] associated with, what was termed at that time, “lymphangiosarcoma.” Therefore, edematous lesions are difficult to clinically diagnose as lymphangiosarcomas without a pathological examination. In the past five decades, these characteristic clinical findings had not adequately been reported, perhaps because most of the reports were published from anatomy and pathology departments [1–4, 20–25].

Lymphatic markers, including VEGFR-3, lymphatic vessel endothelial receptor-1 (LYVE-1), and podoplanin, D2-40, and NZ-1, have recently been reported and used to distinguish lymphatic vessels from blood vessels [6–14]. Immunohistochemical studies using these markers have revealed lymphatic differentiation in angiosarcomas [12, 20–27]. MO-LAS were analyzed using vascular markers using flow cytometry. The results showed similar findings in both MO-LAS and ISO-HAS. Both cell lines are positive for the blood vascular marker, CD31, and the lymphatic markers of VEGFR-3 and podoplanin, including D2-40 and NZ-1. These results indicated that both of these cell lines may have similar characteristics of mixed lymphatic and blood vascular endothelium. Flow cytometric analysis was unable to adequately differentiate between the two cell lines. In point of fact, immunohistochemical studies of tumor tissues failed to complete the differentiation between lymphangiosarcoma and other angiosarcomas [25]. Pathological investigations are limited because they cannot exactly identify these two angiosarcomas.

Finally, the gene expression of *PROX1* was investigated in both cell lines. *PROX1* is a homeobox gene to be expressed in a subpopulation of venous endothelial cells that give rise to the lymphatic system by budding and sprouting [9]. Therefore, in *PROX1* null mice, the development of the lymphatic system is arrested, whereas the cardiovascular system is unaffected [9]. Thus, *PROX1* is regarded as a master gene in the development of the lymphatic system and to identify lymphatic differentiation [28]. This homeobox gene *PROX1* expressed more than 3000-fold in MO-LAS compared with that in ISO-HAS. This result could be definite evidence that MO-LAS is an angiosarcoma cell line of lymphatic origin.

Meanwhile, MO-LAS did not possess notable oncogene expressions nor *p53* mutation (data not shown) as did ISO-HAS [16]. The cytotoxicity assay revealed that MO-LAS was much less sensitive than was ISO-HAS against LAK cells. This suggests that MO-LAS is hardly recognized by the immune system because of less tumor antigenicity. In reconsideration of these results, IL-2 and LAK immunotherapy did not contribute to the patient's prognosis, even though immunotherapy is usually effective for hemangiosarcoma [29–31]. This immunotherapy inefficacy was also observed in our other patients with lymphangiosarcomas.

The establishment of lymphatic differentiated MO-LAS from the patient in the present case with the lymphangiosarcoma may provide evidence confirming the existence of lymphangiosarcoma. MO-LAS could contribute to promoting the investigation of molecular biology for lymphatic differentiation and tumorigenic transformation. Currently, lymphangiosarcoma is more refractory than is hemangiosarcoma, because there are currently no specific therapies for tumors with a lymphatic differentiation [25]. Future studies using MO-LAS will hopefully pave the way for more effective novel therapies for lymphangiosarcoma.
